# Sepsis-associated severe interleukin-6 storm in critical coronavirus disease 2019

**DOI:** 10.1038/s41423-020-00522-6

**Published:** 2020-09-11

**Authors:** Lan Huang, Xuan Zhao, Yu Qi, Hong Li, Guanchao Ye, Yafei Liu, Yi Zhang, Jianjun Gou

**Affiliations:** 1 Biotherapy Center, The First Affiliated Hospital, Zhengzhou University, No. 1 Jianshe East Road, 450052 Zhengzhou, Henan China; 2 Department of Thoracic Surgery, The First Affiliated Hospital, Zhengzhou University, No. 1 Jianshe East Road, 450052 Zhengzhou, Henan China; 3 Department of Clinical Laboratory, The First Affiliated Hospital, Zhengzhou University, No. 1 Jianshe East Road, 450052 Zhengzhou, Henan China

**Keywords:** Prognostic markers, Infection

Coronavirus disease 2019 (COVID-19) is a respiratory disease caused by a novel coronavirus known as severe acute respiratory syndrome coronavirus 2 (SARS-CoV-2).^1,2^ Due to its high transmission and the lack of effective vaccines or antiviral drugs, this novel infectious disease has changed the social and economic lives of human beings in unprecedented ways. Most COVID-19 infections are mild or even asymptomatic, but the poor prognosis of severe or critically ill patients is still a major challenge. The host immune response is a key determinant for viral clearance. Accumulating evidence suggests that typical lymphopenia and cytokine release syndrome are two features that predict poor prognosis in COVID-19.^3^ In particular, the levels of blood interleukin-6 (IL-6) are closely correlated with the severity of COVID-19 and have been considered an independent biomarker for predicting poor prognosis.^4^ However, the sources and dynamics of increased IL-6 in COVID-19 patients remain largely unknown. Here, we reported that septic shock or sepsis was strongly associated with a dramatic rise in blood IL-6 in critical cases and further demonstrated maximal IL-6 levels over a certain threshold as a powerful biomarker for fatal outcomes in COVID-19 patients.

A total of 29 severe or critical patients with confirmed COVID-19 infections were admitted to the First Affiliated Hospital of Zhengzhou University, China. The basic characteristics of these patients are shown in Supplementary Table S1. With the development of COVID-19, the levels of blood cytokines might change dynamically. However, blood samples were collected on admission for the detection of plasma cytokines in most reports.^5^ In this cohort, the frequency of cytokine detection was determined by the treating physicians (range 1–8). The maximal levels of six inflammatory cytokines in COVID-19 patients are shown in Supplementary Table S2. The medians of the maximal plasma IL-2, IL-4, tumor necrosis factor-α (TNF-α), and interferon-γ levels were in the normal range, while the medians of the maximal plasma IL-6 and IL-10 levels were much higher than normal values. Furthermore, the maximal level of plasma IL-10 in the critical group was significantly higher than that in the severe group (median 32.35 pg/ml (interquartile range (IQR) 9.14–49.03) vs. 7.49 pg/ml (IQR 5.95–9.31); *P* = 0.003). Notably, the maximal level of plasma IL-6 was more significantly increased in critical cases than in severe cases (median 1072.98 pg/ml (IQR 453.85–9163.64) vs. 49.14 pg/ml (IQR 27.98–101.30); *P* = 0.001). Interestingly, the levels of IL-6 in four patients in the critical group exceeded the upper limit of detection (14,031 pg/ml) (Fig. 1a). To our knowledge, such high levels of plasma IL-6 have not been reported in COVID-19 patients.

**Fig. 1:**
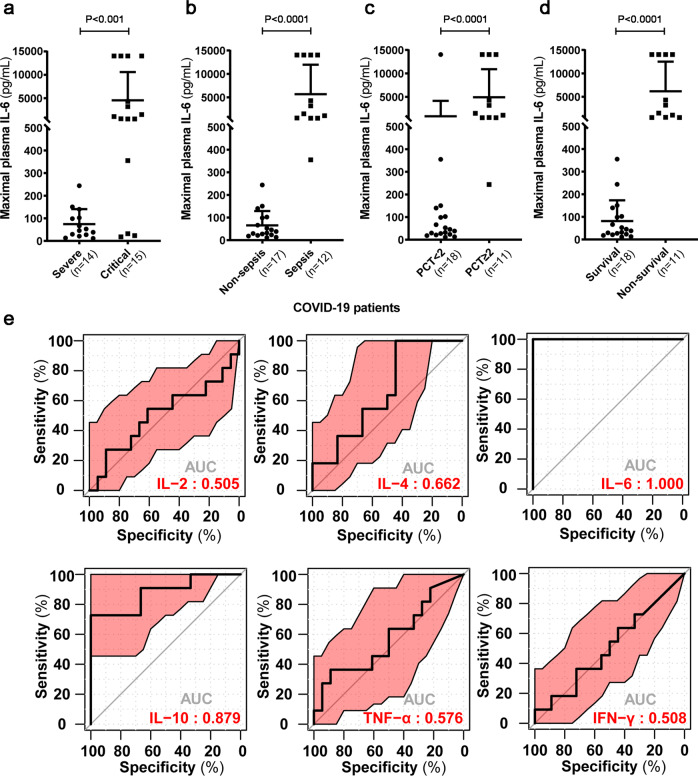
Severe storms of plasma IL-6 were strongly associated with the presence of sepsis and a fatal outcome in 29 severe or critical patients with COVID-19. Comparison of maximal plasma IL-6 levels based on disease severity (**a**), status of sepsis (**b**), PCT value (**c**), and clinical outcome (**d**). The data are presented as the means + SDs. The Mann–Whitney *U* test was applied to assess the statistical significance of the differences between two groups. **e** The prognostic values of maximal plasma cytokines were evaluated by ROC analysis

There are various ways to activate the synthesis of IL-6, such as IL-1β, Toll-like receptors, and the stress response.^6^ To explore the sources of sharply increased IL-6, we carefully reviewed the clinical features of these patients. Septic shock, acute respiratory distress syndrome, and multiple organ dysfunction syndrome were the most common complications in critically ill COVID-19 patients. Although the virus particles of SARS-CoV-2 infect host cells and can induce cytokine storms, IL-6 has also been used as a diagnostic and prognostic biomarker for severe bacterial infections.^7^ Similarly, we found that extremely high levels of IL-6 were strongly associated with the presence of septic shock or sepsis in COVID-19 patients (Fig. 1b). Serum procalcitonin (PCT) is a potential indicator for bacterial infection, and PCT levels above 2 ng/ml are highly suggestive of systemic bacterial infection or sepsis. We further analyzed the relationship between maximal IL-6 and concurrent PCT levels. As expected, maximal plasma IL-6 levels were significantly elevated in COVID-19 patients with high PCT levels compared to patients with low PCT levels (Fig. 1c). Collectively, a dramatic increase in IL-6 secondary to sepsis or other pathological processes rather than the initial viral infection may aggravate severe inflammation and lead to fatal consequences. Thus, clinicians should carefully consider the sources of markedly elevated IL-6 levels.

To investigate the clinical significance of severe IL-6 storms, we analyzed the relationship between maximal plasma IL-6 and the outcomes of COVID-19 patients. Surprisingly, all 11 nonsurvivors had higher levels of maximal IL-6, while survivors had lower levels of maximal IL-6 (Fig. 1d). These results suggested that a certain “threshold” of plasma IL-6 may indicate a risk of fatal outcomes in COVID-19 patients. Therefore, the receiver operating characteristic (ROC) curve of maximal plasma cytokines was used to analyze the prediction efficiency for COVID-19 outcomes. The area under the curve (AUC) of maximal plasma IL-6 was 1.000, indicating a super prediction value for clinical outcomes (Fig. 1e). Furthermore, the optimal cut-off value of maximal IL-6 was 453.85 pg/ml, which had a sensitivity of 100% and a specificity of 100% (Supplementary Table S3). Thus, maximal levels of plasma IL-6 over 453.85 pg/ml could represent the risk of a fatal outcome. Consistently, most nonsurvivors of COVID-19 had a significant prevalence of sepsis or septic shock compared to survivors.^8^ The fatal outcomes of patients with high maximal IL-6 levels indicated that more supervision is required for these patients. Recent studies suggest that IL-6 and TNF-α inhibitor drugs such as tocilizumab may be a potential therapeutic option to ameliorate the negative outcomes of severe COVID-19 patients.^9^ Clinical trials are needed to determine optimal patient selection and timing for the use of tocilizumab.

In conclusion, our findings pointed to host sepsis as contributing to the extraordinary elevation of plasma IL-6 in critically ill patients with COVID-19. The levels of IL-6 should be measured dynamically, as the maximal level of blood IL-6 above a certain threshold may predict a fatal outcome. The dynamic detection of plasma IL-6 is critical for determining the intervention time of an anti-IL-6 receptor antibody.

## Supplementary information

Supplementary Tables
