# Supplementary material for: Sepsis-associated severe interleukin-6 storm in critical coronavirus disease 2019
Source: Cell Mol Immunol. 2020 Sep 11;17(10):1092–4. doi: 10.1038/s41423-020-00522-6 (PMC7484610; doi:10.1038/s41423-020-00522-6)
Supplement: Supplementary file 1 — Supplementary Tables [file 41423_2020_522_MOESM1_ESM.docx]

**Table S1.** Baseline characteristics of 29 severe or critical patients with COVID-19

|  | **Total**  **(n=29)** | **Severe cases**  **(n=14)** | **Critical cases**  **(n=15)** | ***P* value** |
| --- | --- | --- | --- | --- |
| Age (years) | 62.39±14.65 | 57.57±15.29 | 66.40±12.68 | 0.10 |
| ≥50 years, n (%) | 23(79.3%) | 10(61.4%) | 13(86.7%) | 0.39 |
| Sex, n (%) |  |  |  | 0.45 |
| Male | 19(65.5%) | 8(57.1%) | 11(73.3%) |  |
| Female | 10(34.5%) | 6(42.9%) | 4(26.7%) |  |
| Comorbidities, n (%) | 22(75.9%) | 11(78.6%) | 11(73.3%) | 1.00 |
| Hypertension | 12(41.4%) | 9(64.3%) | 3(20.0%) | 0.03 |
| Diabetes | 6(20.7%) | 3(21.4%) | 3(20.0%) | 1.00 |
| Cardiovascular diseases | 5(17.2%) | 2(14.3%) | 3(20.0%) | 1.00 |
| COPD | 8(27.6%) | 2(14.3%) | 6(40.0%) | 0.22 |

Data are means ± SD, n (%) or n/N (%). P values for differences between severe cases and critical cases were calculated by Student t test or χ^2^ test, as appropriate.

COPD, chronic obstructive pulmonary disease; COVID-19, coronavirus disease 2019; SD, standard deviation.

**Table S2.** Maximal plasma cytokine levels of 29 severe or critical patients with COVID-19

|  | **Normal range (pg/ml)** | **Total cases**  **(n=29)** | **Severe cases**  **(n=14)** | **Critical cases**  **(n=15)** | **Z** | ***P* value** |
| --- | --- | --- | --- | --- | --- | --- |
| IL-2 | 0.00-4.13 | 4.03(2.75-6.07) | 4.45(3.15-6.34) | 3.98(2.36-5.65) | -0.808 | 0.419 |
| IL-4 | 0.00-8.37 | 6.96(5.48-11.78) | 11.92(6.33-13.89) | 5.87(5.18-7.68) | -2.531 | 0.011 |
| IL-6 | 0.00-6.61 | 139.76(32.1-1072.98) | 49.14(27.98-101.30) | 1072.98(453.85-9163.64) | -3.190 | 0.001 |
| IL-10 | 0.00-2.31 | 9.18(6.53-32.35) | 7.49(5.95-9.31) | 32.35(9.14-49.03) | -3.011 | 0.003 |
| TNF-α | 0.00-33.27 | 27.37(16.49-61.23) | 29.31(23.61-59.43) | 26.71(11.14-67.73) | -0.481 | 0.630 |
| IFN-γ | 0.00-20.06 | 13.52(2.94-42.03) | 13.48(3.38-40.13) | 13.52(6.24-40.27) | -0.044 | 0.965 |

Data are median (IQR). P values for differences between severe cases and critical cases were calculated by Mann-Whitney U test.

COVID-19, coronavirus disease 2019; IFN-γ, interferon-γ; IL-2, interleukin-2; IL-4, interleukin-4; IL-6, interleukin-6; IL-10, interleukin-10; IQR, interquartile range; TNF-α, tumor necrosis factor-α.

**Table S3.** Predictive performances of maximal plasma cytokine levels for the fatal outcome of 29 severe or critical patients with COVID-19

| **Variables** | **Assessment of validity** | | | | | |
| --- | --- | --- | --- | --- | --- | --- |
|  | **AUC** | **Optimal cut-off value (pg/ml)** | **Sensitivity (%)** | **Specificity (%)** | **Predictive value (%)** | |
|  |  |  |  |  | **Positive** | **Negative** |
| IL-2 | 0.505 | 6.92 | 27.27 | 88.89 | 60.00 | 66.67 |
| IL-4 | 0.662 | 11.57 | 100.00 | 44.44 | 52.38 | 100.00 |
| IL-6 | 1.000 | 453.85 | 100.00 | 100.00 | 100.00 | 100.00 |
| IL-10 | 0.879 | 31.09 | 72.72 | 100.00 | 100.00 | 85.00 |
| TNF-α | 0.576 | 72.77 | 36.36 | 88.89 | 66.67 | 69.57 |
| IFN-γ | 0.508 | 270.24 | 9.09 | 100.00 | 100.00 | 67.29 |

AUC, area under curve; COVID-19, coronavirus disease 2019; IFN-γ, interferon-γ; IL-2, interleukin-2; IL-4, interleukin-4; IL-6, interleukin-6; IL-10, interleukin-10; ROC, receiver operator characteristic; TNF-α, tumor necrosis factor-α.
